# Structural and Immunologic Properties of the Major Soybean Allergen Gly m 4 Causing Anaphylaxis

**DOI:** 10.3390/ijms232315386

**Published:** 2022-12-06

**Authors:** Ekaterina I. Finkina, Ivan V. Bogdanov, Rustam H. Ziganshin, Nikita N. Strokach, Daria N. Melnikova, Ilia Y. Toropygin, Natalia S. Matveevskaya, Tatiana V. Ovchinnikova

**Affiliations:** 1 M.M. Shemyakin & Yu.A. Ovchinnikov Institute of Bioorganic Chemistry, Russian Academy of Sciences, 117997 Moscow, Russia; 2 V.N. Orekhovich Institute of Biomedical Chemistry, Russian Academy of Sciences, 119121 Moscow, Russia; 3 G.N. Gabrichevsky Research Institute for Epidemiology and Microbiology, 125212 Moscow, Russia; 4 Department of Bioorganic Chemistry, Faculty of Biology, Lomonosov Moscow State University, 119234 Moscow, Russia

**Keywords:** Bet v 1 homologue, Gly m 4, soybean allergen, cross-reactivity, gastroduodenal digestion, IgE-binding, lysosomal degradation

## Abstract

Gly m 4 is the major soybean allergen, causing birch pollen cross allergic reactions. In some cases, Gly m 4-mediated anaphylaxis takes place, but the causative factors are still unknown. Here, we studied the structural and immunologic properties of Gly m 4 to shed light on this phenomenon. We showed that Gly m 4 retained its structure and IgE-binding capacity after heating. Gly m 4 was cleaved slowly under nonoptimal gastric conditions mimicking duodenal digestion, and IgE from the sera of allergic patients interacted with the intact allergen rather than with its proteolytic fragments. Similar peptide clusters of Bet v 1 and Gly m 4 were formed during allergen endolysosomal degradation in vitro, but their sequence identity was insignificant. Animal polyclonal anti-Gly m 4 and anti-Bet v 1 IgG weakly cross-reacted with Bet v 1 and Gly m 4, respectively. Thus, we supposed that not only conserved epitopes elicited cross-reactivity with Bet v 1, but also variable epitopes were present in the Gly m 4 structure. Our data suggests that consumption of moderately processed soybean-based drinks may lead to the neutralizing of gastric pH as a result of which intact Gly m 4 can reach the human intestine and cause IgE-mediated system allergic reactions.

## 1. Introduction

The soybean *Glycine max* is a species of legume widely used around the world. In East Asia soybean-based foods such as tofu, miso, or natto are traditionally eaten. Soybeans are a source of proteins in vegetarian diets, soybean sprouts are a rich source of vitamins, and soybean milk is a prevalent substitute for cow’s milk. At the same time, the soybean, along with the peanut, tree nut, wheat, eggs, milk, fish, and shellfish, is one of the most common causes of food allergy [1]. Moreover, soybean is added to various foods, which makes it more difficult to eliminate the allergen from the patient’s diet. As shown, a soybean allergy in most clinical cases is associated with an individual patient’s hypersensitivity, either to highly abundant storage proteins Gly m 5 and Gly m 6, or to Gly m 4, which belongs to the class of homologues of the main birch pollen allergen Bet v 1 [2].

Homologues of Bet v 1 are panallergens involved in the development of pollen–pollen and pollen–food allergic cross-reactions due to their structural similarity and an ubiquitous presence in plant tissues [3]. It is well-known that the birch pollen allergen Bet v 1 is the main sensitizer of this class of allergens, causing cross-reactions in most cases. For example, it has been shown that Bet v 1 was a primary sensitizer in allergy to the apple Mal d 1 and the celery Api g 1 allergens. At the same time, it has been shown that the Bet v 1-independent initiation of food allergy could occur in the case of the carrot Dau c 1 and the hazelnut Cor a 1 [4,5,6]. Today, a role of some clinically significant Bet v 1 homologues from other sources, including the soybean Gly m 4, in the sensitization of the human immune system still remains unclear.

It is known that Gly m 4-mediated allergic reactions as a rule occur in patients with the birch pollen allergy and are associated with the cross-reactivity of specific IgE that can bind both the birch Bet v 1 and the soybean Gly m 4 [7]. Titers of anti-Bet v 1 IgE in the sera of such allergic patients are usually higher than those for Gly m 4 [2,8]. On the other hand, in the sera of birch allergic patients who do not have clinical manifestations of soybean allergy, anti-Gly m 4 IgE are often present [9]. Moreover, as shown by BASALIT trial (double-blind placebo-controlled allergen immunotherapy with rBet v 1-FV in birch-related soya allergy), the subcutaneous injection of the hypoallergenic Bet v 1 variant did not lead to a statistically significant decrease in the level of anti-Gly m 4 IgE in soybean allergic patients and is not particularly effective as an immunotherapy method in this case [10].

Bet v 1-like proteins are generally heat sensitive, denature in an acidic environment, and, consequently, are rapidly degraded by gastric enzymes [11]. Therefore, food allergens of this class cause mostly mild allergic reactions—for example, oral allergic syndrome (OAS) [3,12]. At the same time, life threatening systemic allergic reactions, like urticaria and anaphylaxis, have also been described for some food homologues of Bet v 1, primarily, for the soybean Gly m 4 [13,14]. At this point, the question of how this protein acquires the ability to induce IgE-mediated systemic allergic reactions remains open. Systemic allergic reactions are known to occur after the consumption of moderately processed soybean-based drinks, such as soybean milk, milk shakes, and ice cream, but not after the consumption of soybean foods, which, like other legumes, require long cooking time due to a high content of protease inhibitors [7,8,13,14,15]. Interestingly, the induction of OAS, but not systemic reactions, takes place after consumption of salads containing soybean sprouts [14]. Soybean allergies, especially systemic reactions, are mainly recorded in children, whose digestive systems are not fully formed yet [13], although clinical cases of anaphylaxis in adults have also been described [2,8]. Systemic reactions mediated by other homologues of Bet v 1 have also been reported after the ingestion of raw fruits, vegetables, and nuts [16,17]. A number of factors affecting digestion, such as a plentiful meal, liquid food, fasting, gastric atrophy, and protonic pump inhibitors (PPI) treatment increases the risk of systemic allergic reactions to these proteins [15]. In contrast, soybean milk-induced systemic reactions have been shown to be independent of the PPI treatment [15]. Possibly, a significant increase in gastric pH occurs after consumption of soybean-based liquids having pH of 6.41–7.34 [18].

Previously, we have shown that the allergen Gly m 4 is susceptible to proteolysis under optimal gastric conditions [19]. At the same time, we have demonstrated that the digestibility of Gly m 4 could be significantly reduced upon a change in pH value and pepsin-to-allergen ratio, which are known to be critically dependent upon human age and on the amount and composition of ingested food [20]. Thus, we have proposed that the intact Gly m 4 allergen was able to reach the human intestine under nonoptimal gastric conditions [20]. We also have revealed that the intact soybean allergen Gly m 4 and its proteolytic fragments were able to cross the Caco-2 epithelial barrier and differently induce the production of several anti- and proinflammatory stimuli by immunocompetent cells involved in the Th2 response and sensitization of the immune system [19].

The aim of this study was the further investigation of structural and immunologic properties of Gly m 4 with a view to obtain new data shedding light on the soybean allergen peculiar features that might be responsible for causing severe systemic allergic reactions. Here, we estimated the immunologic similarity of the birch pollen Bet v 1 and the soybean Gly m 4 using animal polyclonal anti-Bet v 1 and anti-Gly m 4 IgG. We studied the effects of heating and different gastroduodenal digestion conditions on the structure and IgE-binding capacity of the soybean allergen. We compared the processing of the birch Bet v 1 and the soybean Gly m 4 in antigen-presenting cells using lysosomal fraction of human macrophages and liquid chromatography combined with tandem mass spectrometry (LC-MS/MS).

## 2. Results and Discussion

### 2.1. Investigation of Immunological Similarity of the Birch Pollen Bet v 1 and the Soybean Gly m 4 by ELISA

**Figure 1:**
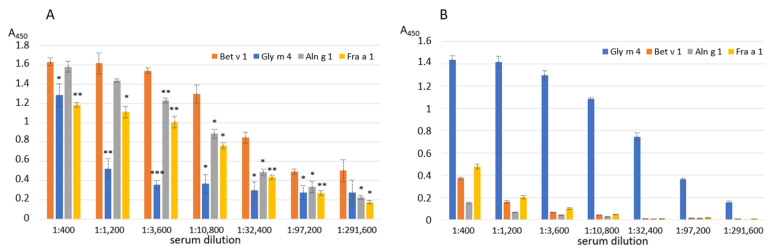
ELISA with the birch Bet v 1, the alder Aln g 1, the soybean Gly m 4 and the strawberry Fra a 1 using polyclonal rabbit anti-Bet v 1 (**A**) or rat anti-Gly m 4 (**B**) IgG. Error bars represent standard deviation between technical replications. Differences in mean A values between Bet v 1 (**A**) or Gly m 4 (**B**) and other allergens were compared by *t*-test (significance levels are: (**A**) * *p* < 0.05, ** *p* < 0.01, *** *p* < 0.005 (**A**); (**B**) *p* < 0.001 for Bet v 1, Aln g 1 and Fra a 3 in all serum dilutions).

The immunological similarity of the birch pollen Bet v 1 and the soybean Gly m 4 was examined in ELISA assays using animal polyclonal anti-Bet v 1 and anti-Gly m 4 IgG. In addition to the recombinant birch Bet v 1 and soybean Gly m 4, two other homologues of Bet v 1, the alder pollen Aln g 1 and the strawberry Fra a 1, were additionally used in comparative experiments. It was shown that the polyclonal rabbit anti-Bet v 1 IgG not only effectively bound to Bet v 1, but also cross-reacted with Aln g 1, and, in a lesser extent, with Fra a 1 (80% and 53% sequence identity with Bet v 1, respectively) (Figure 1A, Supporting Materials, Figure S1). At the same time, only a weak interaction with Gly m 4 (48% sequence identity) was observed (Figure 1A, Supporting Materials, Figure S1). Surprisingly, the polyclonal rat anti-Gly m 4 IgG were characterized by a high specificity and did not interact with any of tested allergens (approximately 50% sequence identity with Gly m 4 structure in the case of all three allergens), except the soybean Gly m 4 (Figure 1B).

Thus, these results argue for an immunologic difference between the soybean Gly m 4 and the birch Bet v 1, possibly not only conservative for Bet v 1-like allergens, but also additional variable epitope regions are present in the Gly m 4 structure. We also hypothesize that this difference may partially explain the lack of a clear positive effect of immunotherapy with the use of the hypoallergenic analogue of Bet v 1 (FV) in patients with a Gly m 4-mediated soybean allergy [10].

### 2.2. Effects of Heating on the Gly m 4 Structure and IgE-Binding Capacity

As mentioned above, Gly m 4-mediated anaphylaxis in birch pollen-related soybean allergic patients is known to occur after the consumption of moderately processed soybean milk [8,13]. The short heating (20 min at 100 °C or shorter heating at higher temperature) of raw soybean milk reduced the content of antinutritional compounds (such as trypsin inhibitors) but allowed it to preserve its nutritional value. At the same time, it is known that heating differently affects the structures of various Bet v 1-like allergens and in some cases leads to a significant decrease in their allergenic potential [21]. Therefore, we decided to test the heating effect on the Gly m 4 structure and its antibody-binding capacity.

**Figure 2:**
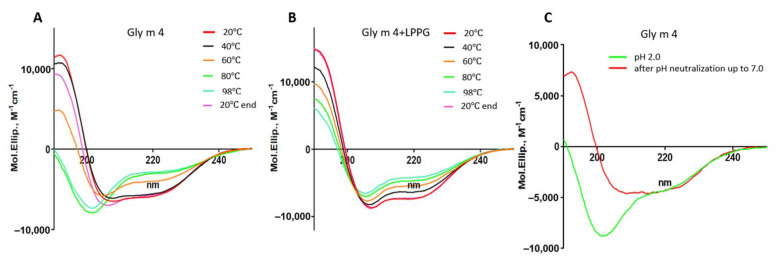
Effects of heating without (**A**) or in the presence of the lysolipid LPPG (at the final concentration of 1 mM) (**B**) and pH change (**C**) on the secondary structure of the soybean allergen Gly m 4. 20 °C end—the CD spectrum of Gly m 4 at 20 °C after cooling down from 98 °C.

The far-UV CD spectrum of Gly m 4 at pH 7.4 revealed a combination of α- and β-secondary structures as we have described previously [20]. Gly m 4 heating up to 60 °C led to the allergen denaturation and its CD spectrum alteration (Figure 2A; Supporting Materials, Table S1). At 98 °C, the protein became unfolded, but after cooling down to 20 °C the CD spectrum took an original shape. The refolding of the Bet v 1 homologue allergens has also been shown previously for the peach Pru p 1 [22] and the celery Api g 1 [21]. At the same time, heating irreversibly destroyed the structures of the apple Mal d 1 and the carrot Dau c 1 [22]. The presence of lysolipid LPPG increased the percentage of α-helices in the Gly m 4 structure, as we have reported previously [20], and decreased a rate of the allergen degradation upon heating at the lipid concentration of 1 mM but had no effect on the concentration of 0.2 mM (Figure 2B; Supporting Materials, Table S1).

**Figure 3:**
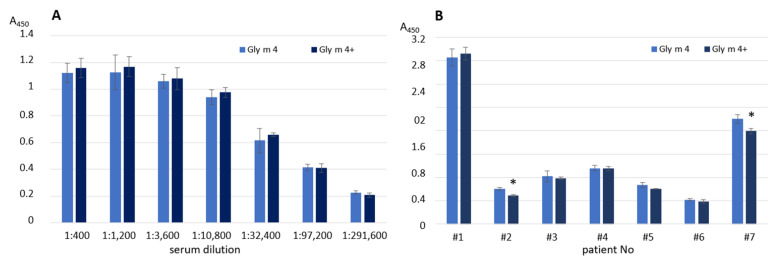
Effects of Gly m 4 heating on the binding of polyclonal rat anti-Gly m 4 IgG (**A**) or human IgE from sera of allergic patients (**B**). Gly m 4, Gly m 4+—the intact and preheated allergen, respectively. Error bars represent standard deviation between technical replications. Differences in mean A values between intact and preheated Gly m 4 for each patient were compared by *t*-test (significance levels is * *p* < 0.05).

The effects of heating on the soybean allergen capacity to bind human IgE from the sera of allergic patients (Supporting Materials, Figure S2, Table S2) as well as polyclonal rat anti-Gly m 4 IgG were examined with ELISA experiments. The allergen preheating during 20 min at 100 °C almost did not affect the antibody-binding capacity of the soybean Gly m 4 in both cases (Figure 3A,B). This observation was in a good agreement with the CD spectroscopy data. A slight decrease of Gly m 4 interaction with IgE was observed with only two sera of allergic patients (Figure 3B). On the contrary, using an immunodot assay, it was shown that the IgE-binding capacity of the thermolabile apple Mal d 1 markedly reduced after incubation for 15 min at 80 °C or after holding for 5 min at 100 °C [17].

Taking into account the data of CD-spectroscopy and results of ELISA experiments, we assumed that the soybean allergen Gly m 4 was able to restore its structure after thermal denaturation during cooking, interact with specific IgE, and induce allergic reactions, up to anaphylaxis.

### 2.3. Effects of Different Gastric pH Values and Pepsin Loadings on Proteolysis of Gly m 4 Mimicking Gastroduodenal Digestion of the Allergen and Its IgE-Binding Capacity

In our previous study we demonstrated that Gly m 4 is very sensitive to pepsin degradation due to its unfolding under strong acidic conditions [20]. The gastric digestion of the soybean allergen significantly reduced upon a change in pH value and pepsin-to-allergen ratio, which probably allowed the intact Gly m 4 to reach the human intestine [20]. We also showed that not only proteolytic fragments, but also the intact Gly m 4 could cross the epithelial barrier at a moderate rate [19]. Here, we investigated how a less acidic pH and lower pepsin-to-allergen ratios mimicking gastric digestion of Gly m 4 in infants as well as in adults after soybean-based liquid food consumption affect its subsequent duodenal degradation and IgE-binding capacity.

At the first stage, using CD spectroscopy, we studied an ability of the soybean allergen to refold after denaturation under highly acidic conditions (Figure 2C; Supporting Materials, Table S1). For that purpose, Gly m 4 was initially dissolved in an HCl solution; after that, the pH of the solution was adjusted from 2.0 to 7.0 by adding of the ammonium bicarbonate buffer. We showed that the refolding of the Gly m 4 structure took place, and a combination of α- and β-secondary structures with a positive maximum at 190 nm and two negative extremes at 206 and 222 nm was observed on the CD-spectrum after pH neutralization.

**Figure 4:**
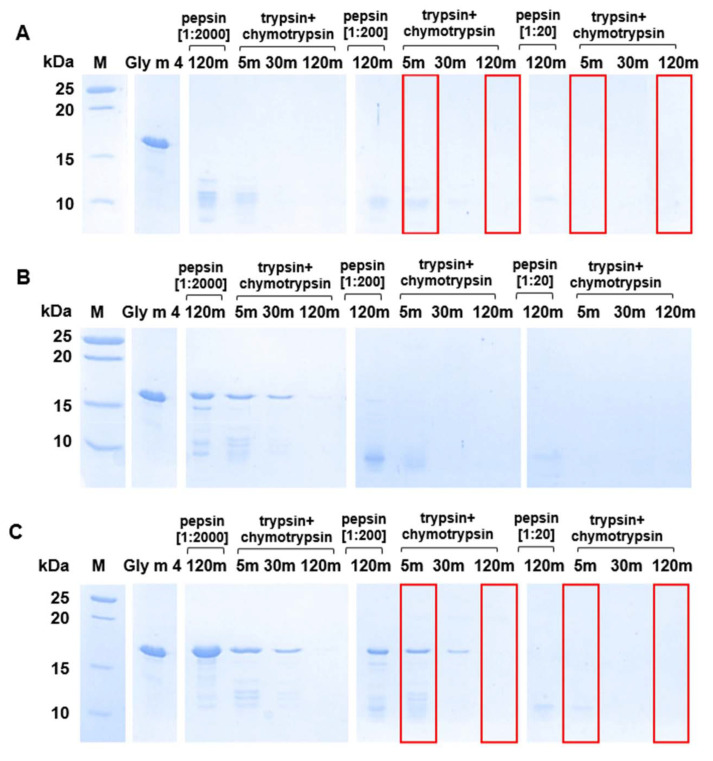
Effects of different gastric pH values ((**A**)—pH 2.0; (**B**)—pH 3.5; (**C**)—pH 5.0) and pepsin loadings (1:2000, 1:200, and 1:20—enzyme-to-allergen mass ratios) on gastroduodenal digestion of Gly m 4, as detected by SDS-PAGE (15%T, 3%C in the separating gel). Gly m 4—the intact allergen; 5 m, 30 m, and 120 m—digestion time, min. M—molecular mass standards. Red frames show Gly m 4 digests which were used in IgE-binding experiments.

Next, proteolytic cleavage mimicking the gastroduodenal digestion of Gly m 4 was simulated at different gastric pH values (pH 2.0, 3.5, and 5.0) and pepsin-to-allergen mass ratios (1:2000, 1:200, and 1:20), while intestine pH as well as trypsin and α-chymotrypsin loadings were the same in all cases. We demonstrated that at pH 2.0, Gly m 4 was quickly degraded by pepsin under different enzyme loadings used as shown by SDS-PAGE (Figure 4A). The proteolytic fragments of Gly m 4 were observed after subsequent allergen degradation with a trypsin/α-chymotrypsin mixture for 5 min only in the case of 1:2000 or 1:200, but not of 1:20 pepsin loading. The rate of gastroduodenal degradation of Gly m 4 was significantly lower at gastric pH 3.5 and especially at pH 5.0 (Figure 4B,C). A trace amount of undigested Gly m 4 along with its proteolytic fragments were observed even after 2 h of the allergen duodenal digestion at pH 3.5 or 5.0 and under pepsin loading of 1:2000. However, under pepsin loading of 1:200, the band corresponding to Gly m 4 was observed after 30 min of duodenal degradation only at gastric pH 5.0. According to densitometric gel analysis, about 20% and 10% of the intact allergen was present in the hydrolysate after 5 and 30 min of duodenal degradation at pH 5.0 under 1:200 pepsin loading, respectively. Only small proteolytic fragments of Gly m 4 were observed after 5 min of duodenal digestion at pH 5.0 and under pepsin loading of 1:20.

Thus, we concluded that the duodenal digestion of Gly m 4, which was not cleaved under gastric conditions, occurred not so quickly, and the intact soybean allergen remained in the intestine for quite a long time and, therefore, could be absorbed through the epithelium and might cause IgE-mediated reactions.

**Figure 5:**
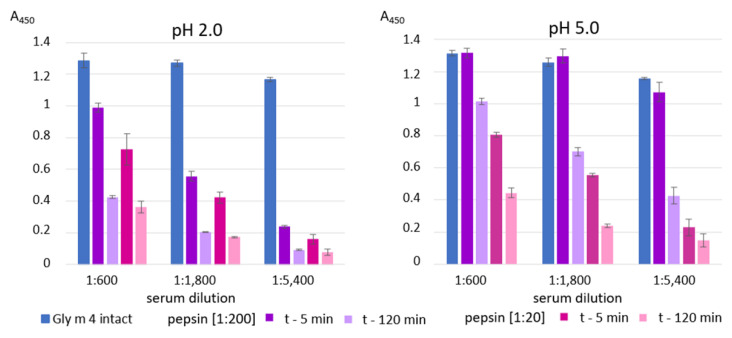
ELISA with the intact Gly m 4 and its gastroduodenal digests obtained at different gastric pH values (pH 2.0 and 5.0) and pepsin-to-allergen mass ratios (1:200 and 1:20) using the polyclonal rat anti-Gly m 4 IgG (t—5 min and t—120 min—time of the allergen digestion with the mixture of trypsin and α-chymotrypsin after Gly m 4 preliminary degradation by pepsin). Error bars represent standard deviation between technical replications.

**Figure 6:**
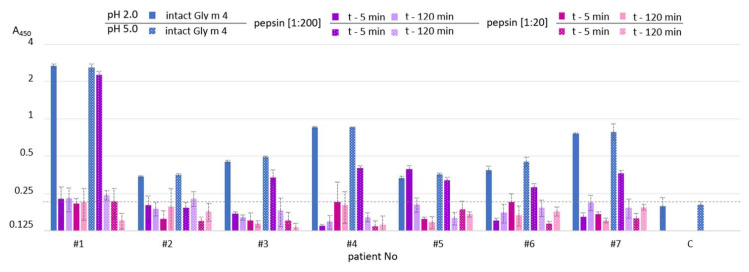
ELISA with the intact Gly m 4 and its gastroduodenal digests obtained at different gastric pH values (pH 2.0 (solid color) and 5.0 (pattern color)) and pepsin-to-allergen mass ratios (1:200 and 1:20) using human IgE from sera of allergic patients (t—5 min and t—120 min—time of the allergen digestion with the mixture of trypsin and α-chymotrypsin after Gly m 4 preliminary degradation by pepsin). C—the control serum from a non-allergic individual. Dotted line shows baseline absorbance corresponding to the control serum. Error bars represent standard deviation between technical replications.

Finally, an ability of Gly m 4 that was digested under different conditions its binding to polyclonal rat anti-Gly m 4 IgG (Figure 5) as well as IgE from the sera of allergic patients (Figure 6) was examined with ELISA. The gastroduodenal digestion of the soybean allergen obtained only at gastric pH 2.0 or 5.0 and under pepsin loading of 1:20 or 1:200 was used in comparative antibody-binding experiments (Figure 4A,C, red frames). In the case of the rat anti-Gly m 4 IgG, a weak antibody binding was observed for hydrolysates obtained at a gastric pH 2.0 or 5.0 and pepsin loading of 1:20 even after only 5 min of subsequent duodenal degradation, most likely due to the presence of only low molecular mass fragments of the allergen in them (Figure 5). For 1:200 pepsin loading, different results were registered for hydrolysates obtained at various gastric pH. Weak antibody binding was also observed for digestion obtained at gastric pH 2.0, even after only 5 min of subsequent duodenal degradation. Surprisingly, the IgG-binding to hydrolysates obtained at gastric pH 5.0 and after 5 min of duodenal degradation was about the same as for the intact Gly m 4, although only 20% of undigested allergen and its large proteolytic fragments were observed on the electrophoregram (Figure 4C, red frames). IgG-binding was also registered under these conditions after duodenal digestion for 120 min.

Rather different results were obtained in ELISA experiments with the sera from allergic patients. As expected, IgE-binding was not observed in the case of almost all sera of allergic patients at gastric pH 2.0 for both pepsin loading and at pH 5.0 for pepsin loading of 1:20 even after 5 min of duodenal degradation (Figure 6). Unlike rat antiserum with a high titer of the anti-Gly m 4 IgG, IgE binding of the digested material obtained at gastric pH 5.0 and pepsin loading of 1:200 after 5 min of duodenal degradation was significantly reduced in most sera of allergic patients. IgE-binding was not registered under these conditions after duodenal digestion during 120 min. Thus, we concluded that human IgE interacted mainly with the intact protein, but not with its fragments.

It was shown with a phage display analysis that three short linear IgE-binding epitope motives are present in the Gly m 4 structure—the dominant motif N_42_VEG_45_, which is highly homologous to the Bet v 1 sequence; N_43_IEG_46_; and two additional motives I_73_DEA_76_ and E_120_TKGD_124_ with low homology to the Bet v 1 sequence [23]. The motif N_43_IEG_46_ is localized in the dominant linear IgE-binding epitope of Bet v 1, which is responsible for the cross-reactivity of Bet v 1 homologues [24]. Previously, we have studied the gastrointestinal digestion of Gly m 4 obtained under simulated optimal gastric conditions (2 h gastric digestion at pH 2.0 and pepsin loading of 1:20, subsequent 2 h duodenal digestion) [19]. The peptide analysis with liquid chromatography combined with tandem mass spectrometry (LC-MS/MS) showed that even in such gastrointestinal digestion of Gly m 4 proteolytic fragments, covering almost complete amino acid sequence of Gly m 4, were present. All the three described linear IgE-binding epitope motifs were present in the structure of proteolytic fragments of the soybean allergen and the most abundant proteolytic fragments of Gly m 4 included the epitope motifs N_42_VEG_45_ and E_120_TKGD_124_. By summing the data obtained here and in our previous work, we assumed that IgE from the sera of allergic patients interacted mainly with conformational, but not with linear, epitopes of the soybean allergen. Therefore, the presence of the intact Gly m 4, but not its proteolytic fragments is probably necessary for the development of IgE-mediated allergic reactions. The consuming of the neutral soybean-based liquid food increases gastric pH and leads to a significant decrease in the rate of Gly m 4 gastric digestion, reaching the intestine with the intact allergen, absorbing through epithelial barrier, and developing IgE-mediated allergic reactions.

### 2.4. Preparation and Characterization of Microsomal or Lysosomal Fractions of Human Macrophages

An endolysosomal degradation assay is widely used for the investigation of allergenic proteins, as it is believed that allergen T-cell epitopes correlate with the peptides resulting from the degradation with endolysosomal extracts isolated from antigen-presenting cells [5]. A total microsomal fraction from antigen-presenting cells is commonly used for that purpose [25]. However, this fraction contains proteins from other cell compartments, the presence of which may affect the results of protein degradation in vitro. To the best of our knowledge, our study is the first attempt to use enriched lysosomal fraction for investigation of allergen endolysosomal degradation in vitro.

Human macrophages, which are considered to be tissue-resident antigen-presenting cells, were used. A number of additional steps for depletion of the mitochondrial fraction were performed for isolation of lysosomes compared with microsomes. An effectiveness of the fractionation was assessed with the measurement of the activity of β-N-acetylglucosaminidase, which is one of the marker enzymes for lysosomes. It was shown that the lysosomal fraction cleaved 4-nitrophenyl-N-acetyl-βD-glucosaminide more effectively than the microsomal fraction (Supporting Materials, Figure S3), which indicated that the former fraction was enriched with lysosomes. Further analysis of the obtained total microsomal and lysosome-enriched fractions was carried out using proteomic tools. The samples were subjected to trypsinolysis and further analyzed with LC-MS/MS. In both, a broad spectrum of proteases was shown to be involved in antigen presentation: cathepsins B, D, H, S, Z, and L [26] (Supporting Materials, Tables S3 and S4). At the same time, β-N-acetylglucosaminidase was detected only in the lysosomal fraction with this method.

**Figure 7:**
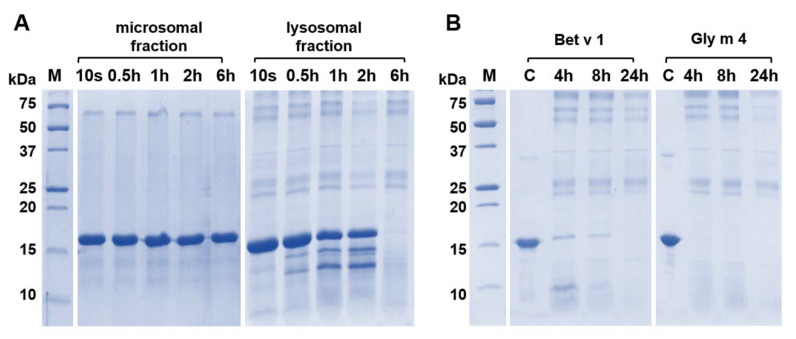
The allergen Gly m 4 degradation in vitro by enzymes of microsomal or lysosomal fractions of human macrophages as detected by SDS-PAGE (15%T, 3%C in separating gel). (**A**) Comparison of rates of the Gly m 4 cleavage in the presence of microsomal or lysosomal fractions for 10 s, 0.5 h, 1 h, 6 h or 24 h. (**B**) Comparison of rates of cleavage of two allergens—the birch Bet v 1 and the soybean Gly m 4 in the presence of the lysosomal fraction; 4 h, 8 h and 24 h—digestion time. M—molecular mass standards. C—the intact Bet v 1 or Gly m 4 (control).

In the next step, we compared the degradation potential of both these fractions. To do this, the soybean Gly m 4 was incubated with equal amounts of the total protein content of microsomal and lysosome-enriched fractions, upon that the samples from the reaction mixture were collected at different time points (0 s, 0.5 h, 1 h, 2 h, and 6 h) and the results were analyzed with SDS-PAGE (Figure 7A). It was shown that the lysosome-enriched fraction had much more pronounced degradation potential, as traces of the fragments were evident during just 30 min. This supports our suggestion that microsomal and lysosomal fractions have different degradation potential in vitro, and we believe that the lysosomal fraction should be used in the endolysosomal degradation assay while studying an allergenic potential of various proteins in vitro.

### 2.5. Comparison of the Birch Bet v 1 and the Soybean Gly m 4 Endolysosomal Degradation

T-cell epitopes of Gly m 4 have not been mapped yet. Here, we performed lysosomal degradation in vitro of both the soybean Gly m 4 and the birch Bet v 1 using the obtained lysosome-enriched fraction to reveal fragments of the soybean allergen resulting from the proteolysis, as well as to compare known T-cell epitopes of Bet v 1 with the resulted Gly m 4 endolysosomal fragments. For that purpose, allergens were incubated with the lysosomal fraction for 4, 8, and 24 h (Figure 7B). Gly m 4 was shown to be more sensitive to the endolysosomal degradation in vitro than Bet v 1, and already after 4 h of incubation, the intact soybean allergen was not detected on the electrophoregram. Moreover, a high sensitivity of Gly m 4 to proteolysis was not associated with the protein unfolding since we have previously shown the absence of the soybean allergen denaturation at pH 5.0 [20].

Today, an endolysosomal degradation rate of allergenic proteins is commonly considered to be linked with their sensitization capacity. However, there is no consensus regarding whether the protein high resistance to proteolysis in experiments in vitro is directly related to its allergenic potential. For instance, it was demonstrated in similar experiments that the celery Api g 1, showing a strong cellular cross-reactivity with Bet v 1, is digested during endolysosomal degradation in vitro much faster than the carrot Dau c 1, for which, on the contrary, the Bet v 1-independent sensitization was proposed [4]. By contrast, the major sensitizer birch pollen Bet v 1.0101 was more sensitive to endolysosomal degradation in vitro than the hypoallergenic isoform Bet v 1.0401 [27].

**Figure 8:**
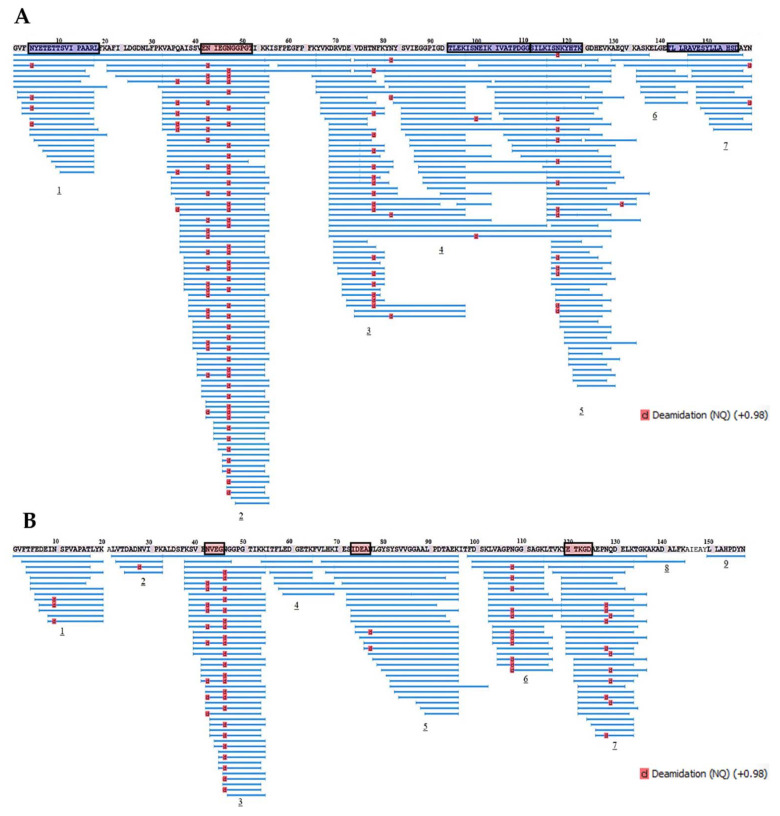
The fragments of Bet v 1 (**A**) and Gly m 4 (**B**) resulted from allergens degradation in vitro by lysosomal enzymes of human macrophages for 8 h. Numbers 1–9 show the main clusters of peptide fragments of the investigated birch and soybean allergens. Dominant IgE-binding linear epitopes of Bet v 1 (E_42_NIEGNGGPGT_52_, accordingly to [29]) and Gly m 4 (N_43_VEG_46_, I_74_DEAN_77_ and E_121_TKGD_125_, accordingly to [23]) are framed in red. Dominant T-cell epitopes of Bet v 1_142–156_ and other epitopes of Bet v 1_4–18,94–111,112–123_, recognized by at least 25% of the patients studied [28], are framed in blue.

**Figure 9:**
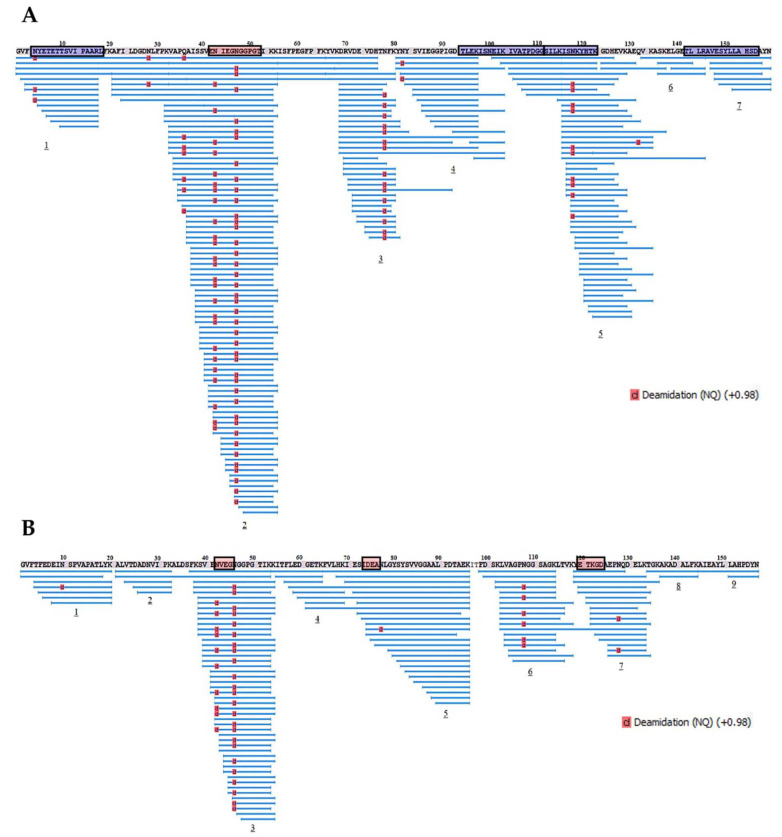
The fragments of Bet v 1 (**A**) and Gly m 4 (**B**) resulted from allergens degradation in vitro by lysosomal enzymes of human macrophages for 24 h. Numbers 1–9 show the main clusters of peptide fragments of the investigated birch and soybean allergens. Dominant IgE-binding linear epitopes of Bet v 1 [29] and Gly m 4 [23] are framed in red. Dominant T-cell epitopes of Bet v 1 [28] are framed in blue.

Further, proteolytic fragments of the birch Bet v 1 and the soybean Gly m 4 resulting from an allergen endolysosomal degradation in vitro for 8 and 24 h and having molecular masses less than 10 kDa were analyzed with LC-MS/MS. The results of Bet v 1 degradation by enzymes of the lysosomal fraction of human macrophages in our experiments were comparable to those previously obtained with the use of the microsomal fraction of monocyte-derived dendritic cells [5] (Figure 8 and Figure 9). At the same time, a greater coverage of the birch allergen structure was noted, possibly owing to a higher enzymatic loading in the enriched lysosomal fraction. Covering almost all amino acid sequences of Bet v 1 and Gly m 4, 7 and 9 fragment clusters, respectively, were found, which allowed the reveal of key sites of the endolysosomal proteolysis (Figure 8 and Figure 9). A decrease in the number of detected peptide fragments of both allergens was observed with an extension of incubation time from 8 to 24 h. It should be noted that a fewer number of Gly m 4 fragments were identified, and they were shorter than those for Bet v 1, possibly due to the greater soybean allergen sensitivity to proteolysis.

All major T-cell activating regions of the birch allergen (the immunodominant epitope Bet v 1_142–156_ and other epitopes Bet v 1_4–18,94–111,112–123_, which were recognized by at least 25% of the patients [28]), were found among the resulted proteolytic fragments of Bet v 1 after its degradation during 8 h. Moreover, all the most represented proteolytic fragments of Bet v 1 particularly contained all above mentioned T-cell epitopes or their parts, except immunodominant Bet v 1_142–156_ (Table 1, Supporting Materials, Table S5). Dominant linear B-cell epitope Bet v 1_42–52_ [29] was also present in the structure of the proteolytic fragments of birch allergen. Almost the same arrangement of peptide clusters was observed for both Bet v 1 and Gly m 4 after their degradation during 8 h as well as 24 h. However, the percentage of sequence identity of the peptide fragments of Bet v 1 and Gly m 4 was relatively low, in particularly: 33%, 59%, 50%, and 60% for T-cell activating regions Bet v 1_4–18,94–111,112–123,142–156_, respectively. Interestingly, all three linear B-cell epitopes of the Gly m 4 sequence (N_43_IEG_46_, I_73_DEA_76_, E_120_TKGD_124_) were present in the structures of the most represented peptide fragments (Table 2, Supporting Materials, Table S6).

Taking into account the higher sensitivity of Gly m 4 to proteolysis, a similar arrangement of peptide clusters formed during endolysosomal degradation of both allergens in vitro, but because of the low sequence identity of these structural regions, some of which can probably be T-cell epitopes of the soybean allergen, we made the following conclusion. One should not exclude the presence of not only cross-reacting with Bet v 1, but also its own T-cell activating regions in Gly m 4 structure, due to which the question about sensitizing potential of soybean allergen remains open.

## 3. Materials and Methods

### 3.1. Materials

Digestive enzymes (porcine pepsin and trypsin, bovine α-chymotrypsin) as well as secondary polyclonal antibodies conjugated to HRP and 3,3′,5,5′-tetramethylbenzidine (TMB) substrates were purchased from Sigma-Aldrich (St. Louis, MO, USA). The recombinant Gly m 4 was overexpressed in *E. coli* and purified as described previously [19]. The recombinant allergens birch Bet v 1, alder Aln g 1 and strawberry Fra a 1 were obtained in a similar way. For that recombinant plasmid, pET-His8-TrxL-Bet v 1/Aln g 1/Fra a 1 were constructed using the sequence encoding the mature Bet v 1.0101 [GeneBank X15877], Aln g 1.0101 [GeneBank S50892], and Fra a 1.0102 [GeneBank AM084674] under the T7 promoter. The recombinant allergens were overexpressed in *E. coli* BL21(DE3)/pET-His8-TrxL-Bet v 1/Aln g 1/Fra a 1 cells and purified as described for Gly m 4 [19]. Homogeneity and the identity of the recombinant allergen samples were confirmed with MALDI mass spectrometry and CD spectroscopy (Supporting Materials, Figure S4).

The sera from patients (n = 70) with pollen and pollen-food allergy from the Moscow region with a higher prevalence allergy to birch were collected on the basis of the Clinical Diagnostic Center of the G.N. Gabrichevsky Research Institute for Epidemiology and Microbiology. The amount of specific IgE (sIgE) to allergen extracts in the patient sera was determined using RIDA qLine Allergy Panel 1–4 (R-Biopharm, Pfungstadt, Germany). The screening of sera containing sIgE to Gly m 4 was performed with an Enzyme-Linked Immuno-sorbent Assay (ELISA). The sera samples from non-allergic individuals were used as a negative control. The sera of 16 allergic patients contained sIgE to Gly m 4 (Supporting Materials, Figure S2, Table S2). Seven sera of Gly m 4^+^ patients in which the content of sIgE was the highest were used in IgE-binding experiments with the intact, preheated, and digested soybean allergen.

### 3.2. CD Spectroscopy

Circular dichroism spectra were recorded at different temperatures using a J-810 spectropolarimeter (jahsco, Hachioji, Tokyo, Japan) in a 0.1 cm path length quartz cell (Hellma GmbH & Co., KG, Mullheim, Germany) in the 190–250 nm range 0.2 using solution of Gly m 4 in 10 mM phosphate buffer, pH 7.4, at a concentration of 30 µM. Lysolipid LPPG (lyso-palmitoyl phosphatidylglycerol) was added at final concentrations of 0.2 or 1 mM. In the case of the pH neutralization experiment, Gly m 4 was dissolved in 0.05 M HCl, pH 2.0, and then the pH of the solution was adjusted to 7 by adding 0.1 M ammonium bicarbonate buffer, pH 8.0.

### 3.3. Gastroduodenal Digestion In Vitro

The gastroduodenal digestion of the recombinant Gly m 4 was simulated as described with some modifications [19,20]. The soybean allergen cleavage mimicking gastric digestion in vitro was performed at pH 2.0, 3.5, or 5.0 for 2 h at 37 °C using 50 ng or 5 ng or 0.5 ng of the porcine pepsin per 1 μg of Gly m 4 (enzyme-to-allergen mass ratio 1:20, 1:200, or 1:2000, respectively). For cleavage mimicking subsequent protein duodenal digestion in vitro, the pH of all mixtures resulting from gastric digestion was adjusted to 7.0 by the addition of 0.1 M ammonium bicarbonate and incubated for 2 h at 37 °C with 2.5 ng of trypsin and 10 ng of α-chymotrypsin per 1 μg of the allergen. The digestion of Gly m 4 was monitored with sodium dodecyl sulfate polyacrylamide gel electrophoresis (SDS-PAGE). Each experiment was carried out three times. A gel analysis was performed using Gel Doc XR^+^ imaging system (Bio-Rad, Hercules, CA, USA) and Image Lab Software.

### 3.4. Antibodies Preparation

The polyclonal anti-Bet v 1 antibodies were prepared by the immunization of two rabbits as we described earlier for lentil Len c 3 allergen in [30]. At the first stage, the rabbits were subcutaneously administered recombinant Bet v 1 (200 μg/rabbit) with complete Freund’s adjuvant. After that, a half dose of the antigen with incomplete Freund’s adjuvant was used twice. The intervals between rabbit immunizations were four weeks.

The polyclonal anti-Gly m 4 antibodies were prepared by the immunization of two rats basically as described in [31]. In this case, the rats were subcutaneously administered recombinant Gly m 4 (250 μg/rat) with complete Freund’s adjuvant. For the next two immunizations, the same dose of the antigen with incomplete Freund’s adjuvant was used. The intervals between rat immunizations were two weeks.

The sera samples obtained from the same animals prior to immunization (pre-immune sera) were used as negative controls (Supporting Materials, Figures S5 and S6).

### 3.5. Immunoglobulin Binding Assay

In the case of ELISA experiments with sera from allergic patients containing sIgE to Gly m 4, the plate wells were coated with the recombinant allergens (0.5 μg/well) in phosphate-buffered saline (PBS), pH 7.4, for 1 h at 37 °C; saturated with 2% bovine serum albumin (BSA, SERVA, Heidelberg, Germany) in PBS buffer for 1 h, at 37 °C; and then incubated with the sera of allergic patients (in 1:4 dilution) overnight at 4 °C. sIgE-binding was detected using the peroxidase-conjugated anti-human IgE from a goat (in 1:2000 dilution) in PBS with 0.5% BSA, and TMB liquid substrate, supersensitive for ELISA. The enzymatic reaction was stopped by 2 N H_2_SO_4,_ and the absorbance values were determined at 450 nm. PBS, containing 0.05% Tween-20 (PBS-T), was used as a washing solution on each step. For sIgE binding experiments with intact, heated at 100 °C for 20 min, or digested (gastric and subsequent duodenal digestion for 5 min or 2 h) Gly m 4, the sera of seven patients containing sIgE to Gly m 4 were selected. Intact, preheated, and digested Gly m 4 samples were coated in wells of 96-well plates (0.5 μg/well), and ELISA was performed as described above. Sera samples from nonallergic individuals were used as a negative control. Each experiment was carried out twice.

The ELISA assays with the polyclonal anti-Bet v 1 rabbit and anti-Gly m 4 rat antisera were performed as described above with some modifications. After blocking free binding sites, the plate wells were incubated with the anti-Bet v 1 rabbit or anti-Gly m 4 rat antisera in PBS (from 1:400 to 1:291,600 serial dilutions) for 2 h at 37 °C. The peroxidase-conjugated anti-rabbit IgG from a goat (in 1:50,000 dilution) or anti-rat IgG from a goat (Invitrogen, Waltham, MA, USA) (in 1:6000 dilution) in PBS with 0.5% BSA were used for detection. Each experiment was carried out twice.

Immunoblotting was performed to confirm the specificity of polyclonal rabbit anti-Bet v 1 and rat anti-Gly m 4 IgG. Following SDS-PAGE, the proteins were electrotransferred to a nitrocellulose membrane (0.45 mkm, Millipore, Burlington, MA, USA) in a buffer containing 20% of methanol and 0.1% of SDS. After washing in PBS, the membranes were blocked for one hour using 2% milk in PBS. After washing in PBS-T, the membrane incubation with polyclonal rabbit anti-Bet v 1 and rat anti-Gly m 4 antibodies in a 0.5% nonfat dry milk solution in PBS in 1:100 dilution during 2 h at 37 °C was carried out. The detection of immune complexes was carried out using relevant secondary antibodies in 1% milk in PBS (in 1:10,000 dilution) and TMB solution for membranes (Supporting Materials, Figure S3).

### 3.6. THP-1 Cell Culture and Macrophage Differentiation

The acute monocytic leukemia THP-1 line (ATCC TIB-202) was cultured in complete RPMI 1640 medium, supplemented with 10% FCS, 1X antibiotic-antimycotic solution, and 20 mM β-mercaptoethanol, in the CO_2_-incubator (5% CO_2_, 37 °C). THP-1 cells were differentiated into immature macrophages (Mφ0) with 100 ng/mL phorbol 12-myristate 13-acetate (Sigma-Aldrich, St. Louis, MO, USA) for 72 h [32].

### 3.7. Subcellular Fractionation of Microsomes and Lysosomes

Microsomal and lysosomal fractions were isolated from Mφ0 by differential centrifugation. All homogenization steps were performed on ice, and centrifugation steps were carried out at +4 °C. For the isolation of microsomal fraction, 10^8^ cells were homogenized in 100 mL of homogenization medium (HM, 10 mM Tris/acetate, 250 mM sucrose, 1 mM Na_2_EDTA, and 1 mM phenylmethylsulfonyl fluoride, pH 7.0) using EmulsiFlex-C3 cell homogenizer (Avestin, Ottawa, ON, Canada) and centrifuged for 10 min at 6000× *g* in a 5810R centrifuge (Eppendorf, Hamburg, Germany). To obtain a total microsomal fraction, the postnuclear supernatant was ultracentrifuged (60 min at 100,000× *g*) in a Beckman SW-27 swinging rotor [27]. For the isolation of purified lysosomal fraction, 10^8^ cells were homogenized in 100 mL of HM using EmulsiFlex-C3 cell homogenizer and centrifuged for 10 min at 2700× *g*. The postnuclear supernatant was then centrifuged in a Beckman SW-27 swinging rotor for 10 min at 20,000× *g*. The supernatant was decanted, and the resulting light mitochondrial pellet (LMP) was suspended in 35 mL HM by 3 to 4 gentle strokes of the pestle of the Dounce homogenizer (Sigma-Aldrich, St. Louis, MO, USA). After centrifugation for 10 min at 20,000× *g* and the decantation of the supernatant, the LMP was resuspended in ∼2 mL HM using a glass rod. The mitochondria were removed from LMP with the incubation of LMP in the presence of 1 mM CaCl_2_ at 37 °C for 5 min [33]. After that, ~2 mL of LMP was loaded gently over 9 mL of 35% Percoll (GE Healthcare, Chicago, IL, USA) in HM and ultracentrifuged for 40 min at 62,000× *g* in a Beckman SW-27 rotor. The fraction containing lysosomes was collected with a syringe, and Percoll was removed using ultracentrifugation for 60 min at 100,000× *g* in a Beckman SW-55 Ti swinging rotor.

Microsomal and lysosomal contents were released with the addition of Triton X-100 (Sigma-Aldrich, St. Louis, MO, USA) at a final concentration of 0.1% with subsequent 5 freeze-thaw cycles on liquid nitrogen and room temperature, respectively. The total protein in each fraction was quantified by Bradford assay in 96-well microplates; the fractions were then aliquoted and stored at −70 °C.

### 3.8. Assay for Determination of Marker Lysosomal Enzyme N-acetyl-β-d-glucosaminidase

The assay was performed in a 96-well microplate in a 0.05 M citrate buffer, pH 5.0, using 1.5 mM 4-nitrophenyl N-acetyl-β-D-glucosaminide as a substrate [34]. The 4-fold dilutions of lysosomal or microsomal fractions containing 3.8, 0.95, or 0.24 μg of total proteins, respectively, were added to the substrate solution and incubated at 37 °C for 5 h. The optical density of solutions in the wells was measured each 30 min at a wavelength of 405 nm.

### 3.9. Allergen Degradation by the Content of Microsomal and Lysosomal Fractions

Degradation assays were performed in 25 µL phosphate-citrate buffer (0.1 M Na_2_HPO_4_, 0.05 M citric acid), pH 5.5, with 2 mM dithiothreitol containing 4 μg of the studied protein, birch Bet v 1 or soybean Gly m 4, and 2 μg of isolated lysosomal or microsomal proteins. Reactions were conducted for 10 s, 0.5 h, 1 h, 2 h, and 6 h at 37 °C and stopped by boiling for 2 min at 100 °C followed by freezing at −70 °C. Sample analysis was carried out using SDS-PAGE. In the case of subsequent LC-MS/MS analysis of hydrolytic fragments of allergens, the aliquots of the reaction mixtures were taken and frozen (−70 °C) after incubation during 8 and 24 h.

### 3.10. LC-MS/MS

In order to identify proteolytic enzymes involved in antigen presentation, in both microsomal and lysosomal fractions of human macrophages, reduction, alkylation, and digestion of the proteins in solution were performed as described previously [35] with minor modifications. A buffer containing 100 mM Tris, pH 8.5, 1% (*w/v*) sodium deoxycholate (SDC), 10 mM Tris(2-carboxyethyl)phosphine (TCEP), and 20 mM 2-chloroacetamide was added to the fraction samples. The samples were heated for 10 min at 95 °C, and the equal volume of trypsin solution in 100 mM Tris, pH 8.5, was added in a 1:100 (*w/w*) ratio. After overnight digestion at 37 °C, peptides were acidified by 1% trifluoroacetic acid (TFA) for SDB-RPS binding, and 20 μg was loaded on three 14-gauge StageTip plugs, equal volume of ethyl acetate was added, and the StageTips were centrifuged at 400× *g*. After washing the StageTips with a 100 μL of 1% TFA/ethyl acetate mixture and 100 μL of 0.2% TFA, peptides were eluted by 50 μL of elution solution contained 50% acetonitrile, 45% water, and 5% ammonia.

To determine the proteolytic fragments of Bet v 1 and Gly m 4 resulted after endolysosomal allergen degradation in vitro during 8 and 24 h, the preparation of the samples for analysis with liquid chromatography combined with tandem mass spectrometry (LC-MS/MS) were carried out as described in [19]. Briefly, the sample solutions (500 µL) were added to 50 µL of 100 mM Tris, pH 8.5, with 1% SDC, heated at 95 °C for 20 min, cooled to 20 °C, and centrifuged at 16,000× *g* for 15 min. The supernatants were transferred into a VIVASPIN spin filter with a 10 kDa MWCO PES membrane preconditioned in the same buffer and centrifuged at 15,000× *g* until the volume reached ~50 µL. The filtrates were collected in a clean tube, washed with 200 µL of 0.5 M NaCl, and acidified with TFA to the final concentration of 1%. The deoxycholic acid precipitate was extracted with ethyl acetate. The peptides contained in the aqueous phase were desalted on Empore SDB-RPS StageTips microcolumns as described above.

All collected material was vacuum-dried and stored at −80 °C. Before analyses, the peptides were dissolved in 2% acetonitrile/0.1% TFA solution and sonicated for 2 min in ultrasonic water bath.

Reverse-phase chromatography was performed with an Ultimate 3000 Nano LC System (Thermo Fisher Scientific, Waltham, MA, USA), which was coupled to the Q Exactive Plus Orbitrap mass spectrometer (Thermo Fisher Scientific, Waltham, MA, USA) via a nanoelectrospray source (Thermo Fisher Scientific, Waltham, MA, USA). The peptides were loaded in loading solution A (0.1% (*v/v*) formic acid, 2% (*v/v*) acetonitrile) and eluted with a linear gradient: 3–35% solution B (0.1% (*v/v*) formic acid, 80% (*v/v*) acetonitrile) for 105 min; 35–55% B for 18 min, 55–99% B for 0.1 min, 99% B during 10 min, 99–2% B for 0.1 min at a flow rate of 500 nL/min. MS1 parameters were as follows: 60 K resolution, 350–2000 scan range, max injection time—30 m/s, AGC target—3 × 10^6^. Ions were isolated with a 1.4 *m/z* window, preferred peptide match and isotope exclusion. Dynamic exclusion was set to 30 s. MS2 fragmentation was carried out in the HCD mode at 17.5 K resolution with the HCD collision energy value of 29%, max injection time—80 m/s, AGC target—1 × 10^5^. Other settings were as follows: charge exclusion—unassigned, 1, >7.

## 4. Conclusions

In the present study, we obtained new data on the structural and immunologic properties of the soybean allergen Gly m 4. We showed that Gly m 4 restored its structure and retained IgE-binding capacity after heating. Under non-optimal gastric conditions, a part of the soybean allergen remained uncleaved, and the subsequent duodenal digestion of Gly m 4 occurred not so quickly. IgE from allergic patients interacted with the intact Gly m 4 rather than with its proteolytic fragments that resulted from gastroduodenal digestion, including those containing linear B-cell epitopes. Taken together, these findings suggest that the development of IgE-mediated system allergic reactions may occur when the intact Gly m 4 can reach the human intestine as a result of nonoptimal conditions of its gastric digestion. This takes place in adults and especially in children after the consumption of moderately processed and neutralizing pH soybean-based drinks.

For the first-time, using the lysosomal fraction of human macrophages, we showed a higher sensitivity of Gly m 4 to proteolysis than that of Bet v 1 in experiments in vitro. Similar clusters of peptide fragments of both allergens were formed during endolysosomal degradation, but a low amino acid sequence identity of these regions, some of which correspond to T-cell epitopes of Bet v 1, was observed. We also demonstrated that anti-Gly m 4 and anti-Bet v 1 IgG from the sera of immunized animals weakly cross-react with the birch Bet v 1 and the soybean Gly m 4 allergens, respectively. All this together argues for the presence of not only conserved epitopes cross-reacting with Bet v 1, but also of variable epitopes in the Gly m 4 structure, which should be considered in the planning of allergen-specific immunotherapy for soybean allergies.

## Figures and Tables

**Table 1:** The most represented proteolytic fragments (peak area > 1.0 × 10^9^) of Bet v 1 identified using LC-MS/MS after the birch allergen Bet v 1 degradation for 8 h in vitro by lysosomal enzymes of human macrophages. Red color shows amino acid sequence of the dominant linear IgE-binding epitope of Bet v 1_42–52_ [29]. Blue color shows fragments of the linear T-cell epitopes of Bet v 1 [28]. *—Deamidation.

Peptide	Amino Acid Residues	−10lgP	Molecular Mass	*m*/*z*	Peak Area
VAPQAISSVENIEGNGGPGTIKK	33–55	50.85	2265.2	1133.6	1.35 × 10^10^
IVATPDGGSILK	104–115	36.56	1169.7	585.8	1.34 × 10^10^
ISNKYHTKGDHEVK	116–129	48.99	1654.8	828.4	1.01 × 10^10^
VAPQAISSVENIEGNGGPGTIK	33–54	53.79	2137.1	1069.6	9.34 × 10^9^
DRVDEVDHTNFK	69–80	44.56	1473.7	737.9	8.62 × 10^9^
SVIEGGPIGDTLEK	84–97	37.60	1413.7	707.9	8.05 × 10^9^
YHTKGDHEVK	120–129	37.56	1212.6	607.3	5.13 × 10^9^
IEGGPIGDTLEK	86–97	40.35	1227.6	614.8	4.50 × 10^9^
TPDGGSILK	107–115	24.76	886.5	444.2	3.52 × 10^9^
NYETETTSVIPAAR	4–17	40.63	1550.8	776.4	2.63 × 10^9^
VIEGGPIGDTLEK	85–97	40.75	1326.7	664.4	2.09 × 10^9^
NKYHTKGDHEVK	118–129	44.99	1454.7	728.4	1.65 × 10^9^
KISFPEGFPFK	55–65	39.27	1295.7	648.9	1.49 × 10^9^
VAPQAISSVENIEGN(+0.98)GGPGTIKK *	33–55	42.90	2266.2	1134.1	1.37 × 10^9^
AISSVENIEGNGGPGTIK	37–54	43.50	1741.9	871.9	1.09 × 10^9^

Peptide—amino acid sequences of the peptides determined by the PEAKS search workflow. A modified residue is followed by a pair of parentheses enclosing the modification mass. −10lgP—the peptide −10lgP score.

**Table 2:** The most represented proteolytic fragments (peak area > 1.0 × 10^9^) of Gly m 4 identified using LC-MS/MS after the soybean allergen degradation for 8 h in vitro by lysosomal enzymes of human macrophages. Red color shows amino acid sequences of three dominant linear IgE-binding epitope fragments of Gly m 4 (N_43_VEG_46_, I_74_DEAN_77_ and E_121_TKGD_125_, accordingly to [23]). *—Deamidation.

Peptide	Amino Acid Residues	−10lgP	Molecular Mass	*m*/*z*	Peak Area
SVENVEGNGGPGTIKK	39–54	46.07	1584.8	793.4	1.91 × 10^10^
SVENVEGN(+0.98)GGPGTIKK *	39–54	44.67	1585.8	397.5	1.17 × 10^10^
KSVENVEGN(+0.98)GGPGTIK *	38–53	19.77	1585.8	529.6	7.07 × 10^9^
VVGGAALPDTAEK	84–96	44.67	1226.7	614.3	6.39 × 10^9^
SVEN(+0.98)VEGNGGPGTIKK *	39–54	33.12	1585.8	793.9	4.63 × 10^9^
ENVEGNGGPGTIKK	41–54	40.12	1398.7	467.2	3.00 × 10^9^
NVEGNGGPGTIK	42–53	29.76	1141.6	571.8	2.01 × 10^9^
IESIDEANLGYSYSVVGGAALPDTAEK	70–96	59.08	2768.3	1385.2	1.75 × 10^9^
NVEGN(+0.98)GGPGTIKK *	42–54	37.41	1270.7	424.6	1.64 × 10^9^
ETKGDAEPNQDELK	120–133	51.28	1572.7	787.4	1.51 × 10^9^
TKGDAEPNQDELK	121–133	50.34	1443.7	722.9	1.49 × 10^9^
IDEANLGYSYSVVGGAALPDTAEK	73–96	55.12	2439.2	1220.6	1.22 × 10^9^
YETKGDAEPNQDELK	119–133	51.19	1735.8	868.9	1.21 × 10^9^

Peptide—amino acid sequences of the peptides determined by the PEAKS search workflow. A modified residue is followed by a pair of parentheses enclosing the modification mass. −10lgP—the peptide −10lgP score.

## Data Availability

All data generated and analyzed during this study are included in this published article and its Supplementary Materials File.

## Supplementary Materials

The following supporting information can be downloaded at: https://www.mdpi.com/article/10.3390/ijms232315386/s1.
