# Supplementary material for: Structural and Immunologic Properties of the Major Soybean Allergen Gly m 4 Causing Anaphylaxis
Source: Int J Mol Sci. 2022 Dec 6;23(23):15386. doi: 10.3390/ijms232315386 (PMC9736301; doi:10.3390/ijms232315386)
Supplement: Supplementary file 1 [file ijms-23-15386-s001.zip › ijms-2027339-supplementary.pdf]

Supporting information

Table S1. Secondary structure estimation (%) predicted from far-UV CD spectra.

sample	conditions	α-helix, %	β-sheet, %	β-turn, %	random, %	NRMSD*
Gly m 4	pH 2.0	10.0	31.5	22.8	35.5	0.02
	after pH neutralization up to 7.0	14.2	32.9	21.4	31.5	0.02
Gly m 4 (pH 7.4)	20 °C	19.0	30.6	20.8	29.7	0.02
	98.5 °C	7.6	35.3	23.1	34.1	0.04
	20 °C end	18.0	29.3	21.2	31.5	0.03
Gly m 4 + 0.2 mM LPPG (pH 7.4)	20 °C	21.2	29.0	19.8	30.1	0.02
	98.5 °C	6.9	36.0	22.6	34.6	0.04
	20 °C end	19.4	29.6	20.6	30.3	0.02
Gly m 4 + 1 mM LPPG (pH 7.4)	20 °C	23.7	25.4	20.3	30.6	0.02
	98.5 °C	16.2	33.0	21.2	29.6	0.03
	20 °C end	23.1	25.5	20.7	30.8	0.02

Table S2. Characterization of patient sera containing sIgE to Gly m 4.

No.	Age (y.o.)	Sex (M/F)	sIgE to birch		sIgE to alder		sIgE to soybean		Another allergen source
			IU/ml	RAST class	IU/ml	RAST class	IU/ml	RAST class	
1	5	M	>100	6	nd	nd	1.04	2.1	ct, wt, hz, p
2	4	M	>100	6	>100	6	1.25	2.1	H, G, ct, wt, hz, p, po, wn, am
3	4	M	>100	6	55.12	5.1	nd	nd	H, G
4	5	M	>100	6	nd	nd	0.15	0.4	G, ct, wt, p, hz
5	6	M	>100	6	8.95	3.3	nd	nd	H, hz
6	5	M	>100	6	>100	6	nd	nd	H, G, O
7	5	M	75.31	5.5	14.15	3.7	2.68	2.7	H, G, p, wh, ct, hz
8	2	M	>100	6	>100	6	nd	nd	H
9	7	M	45.95	4.8	7.92	3.3	nd	nd	H
10	4	M	12.86	3.6	0.73	2	nd	nd	H, G
11	3	M	>100	6	>100	6	1.67	2.3	H, G, O, hz,ct, wh, cl, ap, wn, am, po
12	7	M	>100	6	9.2	3.4	nd	nd	H, hz
13	6	F	40 .64	4.7	9.7	3.4	nd	nd	H
14	6	M	>100	6	85.39	5.7	nd	nd	H, G
15	9	M	90.88	5.8	16.74	3.9	nd	nd	H, G, hz
16	6	M	>100	6	nd	nd	nd	nd	hz

nd – not determined.

Description of RAST classes: 0 class – not detectable or trace IgE level [0,00-0,34 IU/ml]; 1 class – threshold level [0,35-0,69 IU/ml]; 2 class – elevated IgE level [0,70-3,49 IU/ml]; 3 class - significantly elevated IgE level [3,50-17,49 IU/ml]; 4 class – high IgE level [17,5-49,99 IU/ml]; 5 class – very high IgE level [50,0-99,99 IU/ml]; 6 class - extremely high IgE level [⩾100,0 IU/ml]

Inhalant: B = birch pollen, G = grass pollen, A = alder pollen, H = hazel pollen, O=oak pollen

Food: ct = carrot, po = potato, wt = wheat, hz = hazelnut, am = almond, wn = walnut, ry = rye, cl = celery, ap = apple; p=peanut

Table S3. Profiles of lysosomal proteolytic enzymes detected by LC-MS/MS in microsomal fraction of human macrophages.

Accession	-10lgP	Coverage (%)	Area Sample	Number of peptides	Number of unique peptides	Description
P07858	228.1	37	3.53×10^{8}	13	13	Cathepsin B
P29144	296.01	38	2.84×10^{8}	36	36	Tripeptidyl-peptidase 2
P53634	192.71	21	2.74×10^{8}	10	10	Dipeptidyl peptidase 1
P15144	260.81	36	2.63×10^{8}	30	15	Aminopeptidase N
P25774	202.79	41	1.98×10^{8}	11	11	Cathepsin S
P07339	192.98	36	1.41×10^{8}	11	11	Cathepsin D
O75976	236.94	24	1.23×10^{8}	27	27	Carboxypeptidase D
Q9UBR2	182.3	42	6.36×10^{7}	8	8	Cathepsin Z
P07711	102.68	18	3.04×10^{7}	5	5	Cathepsin L1
P09668	108.79	20	1.33×10^{7}	4	4	Pro-cathepsin H

-10lgP—the peptide -10lgP score.

Table S4. Profiles of lysosomal proteolytic enzymes detected by LC-MS/MS in lysosomal fraction of human macrophages.

Accession	-10lgP	Coverage (%)	Area Sample	Number of peptides	Number of unique peptides	Description
P15144	297.44	46	2.03×10^{9}	46	17	Aminopeptidase N
P53634	200.66	34	2.54×10^{8}	11	11	Dipeptidyl peptidase 1
P07339	185.03	49	2.27×10^{8}	16	16	Cathepsin D
P25774	162.85	45	9.94×10^{7}	12	12	Cathepsin S
P07858	185.31	42	8.69×10^{7}	11	11	Cathepsin B
P07711	184.82	52	6.99×10^{7}	10	10	Cathepsin L1
P29144	116.59	8	3.22×10^{7}	7	7	Tripeptidyl-peptidase 2
Q9UBR2	73.71	19	1.35×10^{7}	4	4	Cathepsin Z
O75976	109.32	6	6.65×10^{6}	6	6	Carboxypeptidase D
P09668	62.67	7	3.61×10^{6}	2	2	Pro-cathepsin H

-10lgP—the peptide -10lgP score.

Table S5. The most represented proteolytic fragments (peak area >1.0×10⁹) of Bet v 1 identified using LC-MS/MS after birch allergen degradation during 24 h *in vitro* by lysosomal enzymes of human macrophages. Red color shows amino acids from dominant linear IgE-binding epitope of Bet v $1_{42\text{-}52}$ [29]. Blue color shows fragments of the linear T-cell epitopes of Bet v 1 [30]. * - Deamidation.

Peptide	Amino acid number	-10lgP	Molecular weight	*m/z*	Peak area
DRVDEVDHTNFK	69-80	44.08	1473.7	737.9	1.52×10^{10}
VAPQAISSVENIEGNGGPGTIKK	33-55	53.53	2265.2	1133.6	1.24×10^{10}
KVAPQAISSVENIEGNGGPGTIK	32-54	49.03	2265.2	756.1	1.09×10^{10}
TPDGGSILK	107-115	30.68	886.5	444.2	9.36×10^{9}
DRVDEVDH	69-76	31.31	983.4	492.7	6.08×10^{9}
VAPQAISSVENIEGNGGPGTIK	33-54	51.92	2137.1	1069.6	5.94×10^{9}
YHTKGDHEVK	120-129	39.06	1212.6	607.3	5.46×10^{9}
GPIGDTLEK	89-97	26.61	928.5	465.3	5.13×10^{9}
IVATPDGGSILK	104-115	30.сен	1169.7	585.8	4.10×10^{9}
ISNKYHTKGDHEVK	116-129	51.97	1654.8	828.4	2.10×10^{9}
IEGGPIGDTLEK	86-97	33.62	1227.6	614.8	1.95×10^{9}
VENIEGNGGPGTIK	41-54	38.42	1383.7	692.9	1.31×10^{9}
AISSVENIEGNGGPGTIK	37-54	45.59	1741.9	871.9	1.27×10^{9}
DEVDHTNFK	72-80	38.29	1103.5	552.8	1.04×10^{9}

Peptide—amino acid sequences of the peptides determined by the PEAKS search workflow. -10lgP—the peptide -10lgP score.

Table S6. The most represented proteolytic fragments (peak area >1.0×10⁹) of Gly m 4 identified using LC-MS/MS after soybean allergen degradation during 24 h *in vitro* by lysosomal enzymes of human macrophages. Red color shows amino acids from three dominant linear IgE-binding epitope fragments of Gly m 4 ($N_{43}VEG_{46}$, $I_{74}DEAN_{77}$ and $E_{121}TKGD_{125}$, accordingly to [23]). * - Deamidation.

Peptide	Amino acid number	-10lgP	Molecular weight	m/z	Peak area
VVGGAALPDTAEK	84-96	45.06	1226.6	614.3	1.08×10^{10}
SVENVEGNGGPGTIKK	39-54	41.55	1584.8	793.4	8.76×10^{9}
NVEGNGGPGTIKK	42-54	37.21	1269.7	424.2	5.29×10^{9}
ENVEGNGGPGTIKK	41-54	44.74	1398.7	467.2	4.77×10^{9}
SVENVEGN(+0.98)GGPGTIKK*	39-54	44.08	1585.8	793.9	2.88×10^{9}
NVEGN(+0.98)GGPGTIK*	42-53	38.01	1142.6	572.3	2.72×10^{9}
ENVEGN(+0.98)GGPGTIK*	41-53	36.29	1271.6	424.9	1.53×10^{9}

Peptide—amino acid sequences of the peptides determined by the PEAKS search workflow. A modified residue is followed by a pair of parentheses enclosing the modification mass. -10lgP—the peptide -10lgP score.

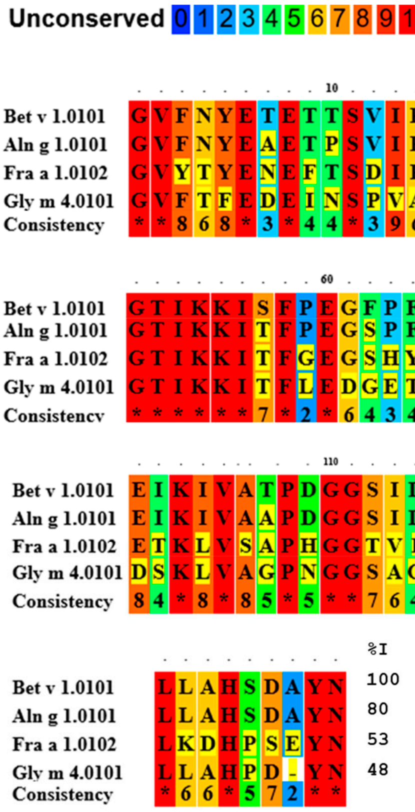

Figure S1. Sequence alignment of pollen (birch Bet v 1, alder Aln g 1) and food (soybean Gly m 4, strawberry Fra a 1) allergens of the Bet v 1 homologue family. Color transition from blue to red shows an increase in the conservation of amino acid residues. Amino acids of the allergens that differ from those of the major sensitizer of this family, the birch pollen Bet v 1, are highlighted in yellow. %I, percentage of sequence identity.

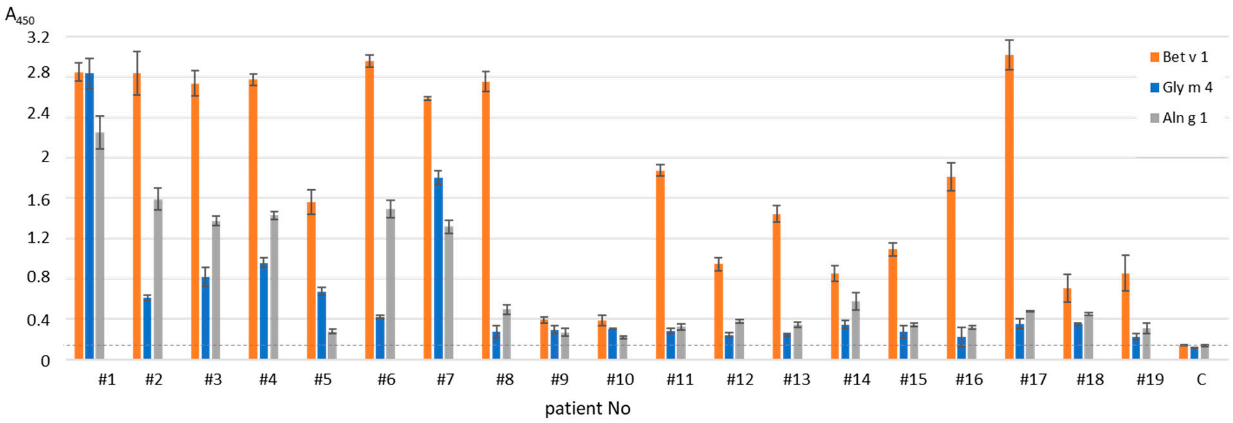

Figure S2. ELISA with birch Bet v 1, alder Aln g 1 and soybean Gly m 4 using the patient sera containing sIgE to these allergens. C – control serum from non-allergic individual. Dotted line shows baseline absorbance corresponding to control serum. Error bars represent standard deviation between technical replications.

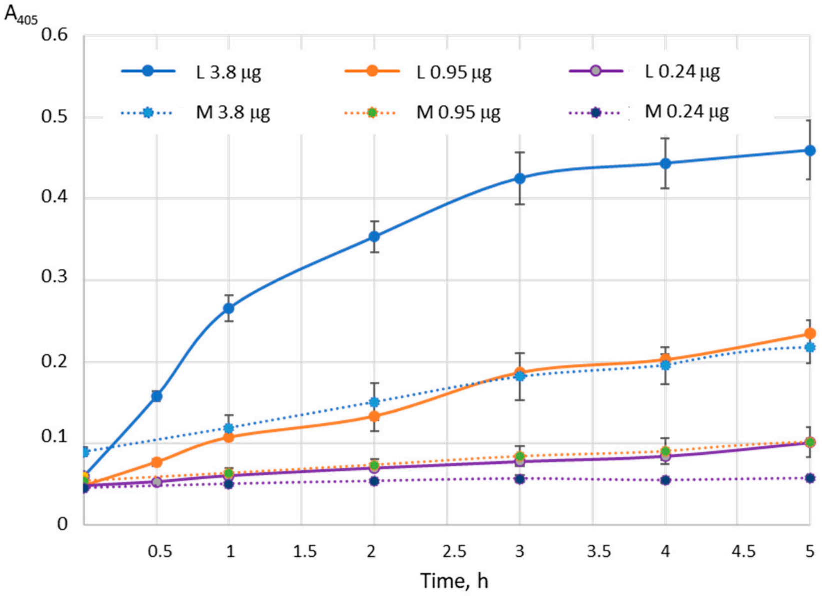

Figure S3. Cleavage of 4-nitrophenyl-N-acetyl-β-D-glucosaminide, which is a substrate of one of the marker enzymes of lysosomes, N-acetyl-glucosaminidase, in the presence of microsomal (M) and lysosomal (L) fractions of human macrophages. Error bars represent standard deviation between technical replications.

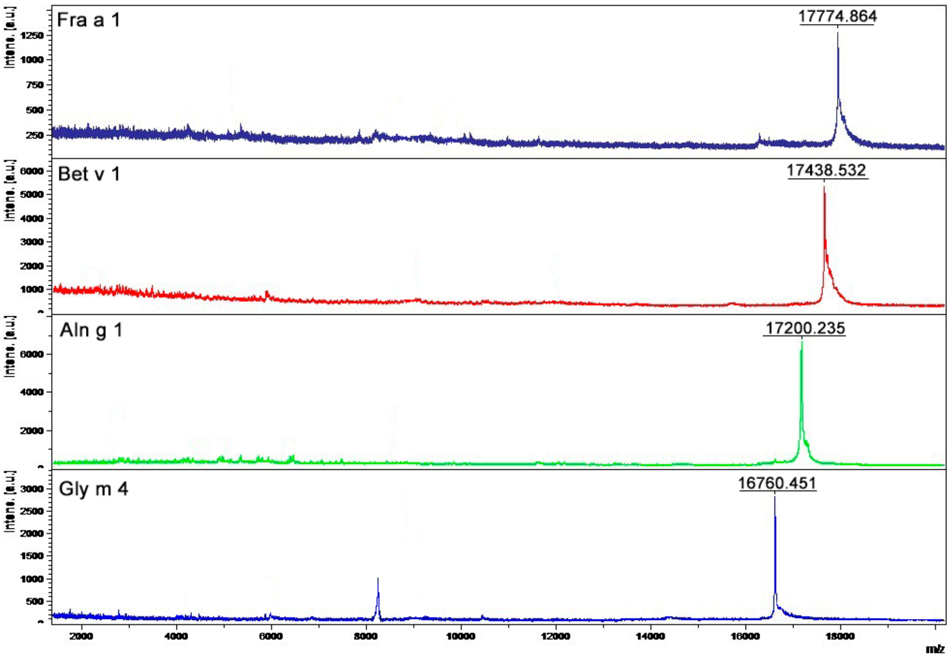

Figure S4. MALDI mass spectra of recombinant allergens.

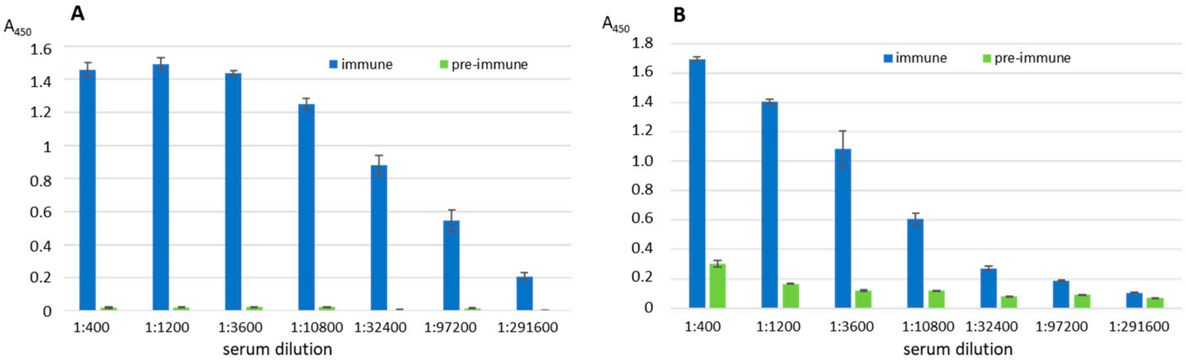

Figure S5. ELISA analysis of polyclonal rat anti-Gly m 4 (A) and rabbit anti-Bet v 1 (B) antisera using soybean Gly m 4 (0.5µg/well) or birch Bet v 1 (0.5µg/well) as an antigen, respectively. Pre-immune sera of animals are used as negative controls. Error bars represent standard deviation between technical replications.

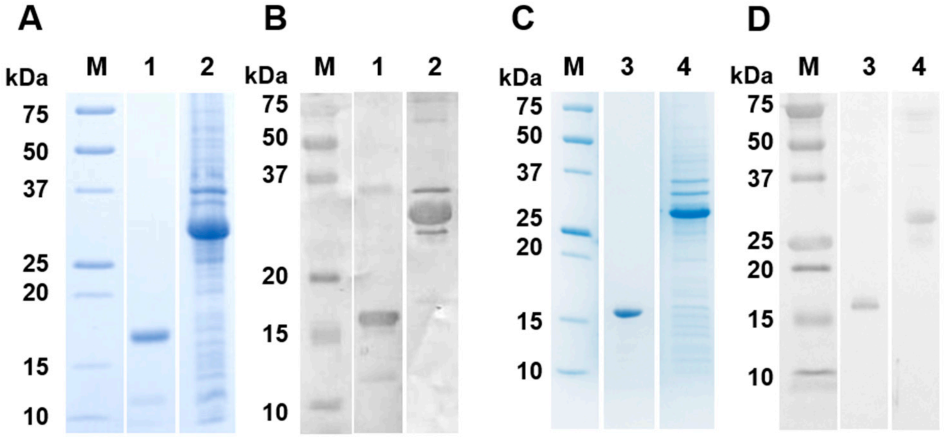

Figure S6. Western blot analyses of polyclonal rabbit anti-Bet v 1 (A, B) and rat anti-Gly m 4 (C, D) antibodies. (A, C) – SDS-PAGE; (B, D) – immublotting with relevant antibodies. 1, 3 – recombinant birch Bet v 1 or soybean Gly m 4, respectively; 2, 4 – cell lysate of *E. coli* BL21(DE3)/pET-His8-TrxL-Bet v 1 or BL21(DE3)/pET-His8-TrxL-Gly m 4, respectively, after induction of the protein expression by isopropyl β-D-1-thiogalactopyranoside. M – molecular mass standards.
